# Particulate Matter, DNA Methylation in Nitric Oxide Synthase, and Childhood Respiratory Disease

**DOI:** 10.1289/ehp.1104439

**Published:** 2012-05-16

**Authors:** Carrie V. Breton, Muhammad T. Salam, Xinhui Wang, Hyang-Min Byun, Kimberly D. Siegmund, Frank D. Gilliland

**Affiliations:** 1 Department of Preventive Medicine, Keck School of Medicine, University of Southern California, Los Angeles, California, USA; 2 Exposure, Epidemiology and Risk Program, Harvard School of Public Health, Boston, Massachusetts, USA

**Keywords:** air pollution, asthma, DNA methylation, epigenetics, PM_2.5_, wheeze

## Abstract

Background: Air pollutants have been associated with childhood asthma and wheeze. Epigenetic regulation of nitric oxide synthase—the gene responsible for nitric oxide production—may be affected by air pollutants and contribute to the pathogenesis of asthma and wheeze.

Objective: Our goal was to investigate the association between air pollutants, DNA methylation, and respiratory outcomes in children.

Methods: Given residential address and buccal sample collection date, we estimated 7-day, 1-month, 6-month, and 1-year cumulative average PM_2.5_ and PM_10_ (particulate matter ≤ 2.5 and ≤ 10 µm aerodynamic diameter, respectively) exposures for 940 participants in the Children’s Health Study. Methylation of 12 CpG sites in three *NOS* (nitric oxide synthase) genes was measured using a bisulfite-polymerase chain reaction Pyrosequencing assay. Beta regression models were used to estimate associations between air pollutants, percent DNA methylation, and respiratory outcomes.

Results: A 5-µg/m^3^ increase in PM_2.5_ was associated with a 0.20% [95% confidence interval (CI): –0.32, –0.07] to 1.0% (95% CI: –1.61, –0.56) lower DNA methylation at *NOS2A* position 1, 0.06% (95% CI: –0.18, 0.06) to 0.58% (95% CI: –1.13, –0.02) lower methylation at position 2, and 0.34% (95% CI: –0.57, –0.11) to 0.89% (95% CI: –1.57, –0.21) lower methylation at position 3, depending on the length of exposure and CpG locus. One-year PM_2.5_ exposure was associated with 0.33% (95% CI: 0.01, 0.65) higher in average DNA methylation of 4 loci in the *NOS2A* CpG island. A 5-µg/m^3^ increase in 7-day and 1-year PM_2.5_ was associated with 0.6% (95% CI: 0.13, 0.99) and 2.8% (95% CI: 1.77, 3.75) higher *NOS3* DNA methylation. No associations were observed for *NOS1*. PM_10_ showed similar but weaker associations with DNA methylation in these genes.

Conclusions: PM_2.5_ exposure was associated with percent DNA methylation of several CpG loci in NOS genes, suggesting an epigenetic mechanism through which these pollutants may alter production of nitric oxide.

Air pollution exposures have been implicated as important risk factors for respiratory health. Air pollution has been associated with decreases in lung function growth in childhood and lung function level in adulthood, asthma exacerbation and onset, and asthma symptoms (Ackermann-Liebrich et al. 1997; Forbes et al. 2009; Gauderman et al. 2004, 2007; Islam et al. 2007; McConnell et al. 2003, 2010; Peters et al. 1999; Sarnat and Holguin 2007). One way in which air pollution, and PM_2.5_ (particulate matter ≤ 2.5 µm aerodynamic diameter) in particular, might affect health outcomes is by altering nitric oxide homeostasis, an important player in the modulation of airway and vascular smooth muscle tone and inflammation (Batra et al. 2007). Nitrosative stress and regulation of nitric oxide play a key role in the pathophysiology of allergic airway diseases (Fitzpatrick et al. 2009; Sugiura et al. 2008). For example, fractional concentration of exhaled nitric oxide (Fe_NO_) is measurably higher in children with eosinophilic airway inflammation and active asthma or allergic airway diseases, conditions in which airway inflammation plays a prominent role (Bastain et al. 2011; Fitzpatrick et al. 2009; Pijnenburg and De Jongste 2008). Short term PM exposure is also associated with higher Fe_NO_ (Berhane et al. 2011).

Production of nitric oxide (NO) is regulated via the nitric oxide synthase pathway. NO is synthesized from l-arginine by three NO synthase (NOS) isoforms, neuronal NOS (nNOS; encoded by *NOS1*), inducible NOS (iNOS; encoded by *NOS2A*), and endothelial NOS (eNOS; encoded by *NOS3*). The availability of intracellular l-arginine is a rate-limiting factor in NO production (Morris 2004). All three NOS isoforms are expressed in airway epithelium (Ricciardolo et al. 2006; Sheffield et al. 2006; Shimokawa and Tsutsui 2010).

Epigenetic variation in NOS genes may alter NO synthesis by affecting NOS function or expression. These changes, in turn, have the potential to influence respiratory health outcomes. *In vitro* and animal studies have demonstrated that epigenetic changes, including DNA methylation and histone modifications in both *iNOS* and *eNOS*, are associated with gene expression (Chan GC et al. 2005; Chan Y et al. 2004; Xu et al. 2010). Thus, existing evidence suggests that abnormal epigenetic variation in the NOS system might perturb NO homeostasis in a manner that adversely affects respiratory health.

Studies in humans are beginning to suggest that air pollution exposure is associated with DNA methylation. Traffic particles were associated with a decrease in DNA methylation levels in LINE-1 (long interspersed nuclear element-1) and *Alu* (short interspersed nuclear element) repeat-elements in a cohort of elderly men (Baccarelli et al. 2009; Madrigano et al. 2011), and decreased promoter methylation in *NOS2A* was observed in a group of steel workers after a 3-day work shift (Tarantini et al. 2009). Children living in more highly polluted communities had changes in DNA methylation of *FOXP3* (Nadeau et al. 2010) and *ACSL3* (Perera et al. 2009) genes, both important in asthma morbidity and symptoms in children. Thus, emerging evidence suggests a link between air pollution exposures, alterations in DNA methylation levels, and downstream health outcomes.

In the present study, we investigated whether short- and long-term exposure to PM_2.5_ and PM_10_ (particulate matter ≤ 10 µm aerodynamic diameter) were associated with DNA methylation levels at CpG loci in the NOS genes. CpG loci were selected in the promoter regions of each gene based on previous evidence from Tarantini et al. (2009) or an increased likelihood for affecting gene expression or transcription based on a gene search using the UCSC genome browser (http://genome.ucsc.edu/), ENCODE data on DNase hypersensitivity (http://genome.ucsc.edu/ENCODE/downloads.html), and transcription factor binding sites. We also evaluated whether DNA methylation in these genes was associated with asthma and wheeze. We tested these hypotheses in a population-based study of children who had participated in the southern California Children’s Health Study (CHS).

## Methods

*Study population.* This study was nested in the ongoing CHS (McConnell et al. 2006). We sampled children from the 5,341 kindergarteners and first-graders who were enrolled in the study in 2002. A subset of 940 non-Hispanic white and Hispanic white children who had buccal samples collected, genetic data available, and Fe_NO_ measurements collected were selected for DNA methylation analysis, as described previously (Breton et al. 2011).

Parents or legal guardians provided written informed consent for all study subjects. The Institutional Review Board of the University of Southern California approved this study.

*Buccal sample collection and processing.* Children were provided with two toothbrushes and instructed to brush their teeth with the first one. They were instructed to gently brush the buccal mucosa with the second toothbrush. The brush was then placed in a leak proof container that was filled with an alcohol-based fixative. Children then swished liquid throughout their mouths and expelled the fluid into a container. Most buccal-cell specimens were collected at school under the supervision of study staff. The remaining specimens were collected at home and sent to us by mail.

Buccal-cell suspensions were centrifuged at 2,000 × *g* on the day they were received in the laboratory. The pellets were stored frozen at –80°C until used for DNA extraction, at which time they were resuspended and incubated in 600 μL lysis solution from a PUREGENE DNA isolation kit (#D-5000; GENTRA, Minneapolis, MN) containing 100 μg/mL proteinase K overnight at 55°C. DNA extraction was performed according to manufacturer’s recommendations. The DNA samples were resuspended in the hydration solution (GENTRA) and stored at –80°C.

**Figure 1:**
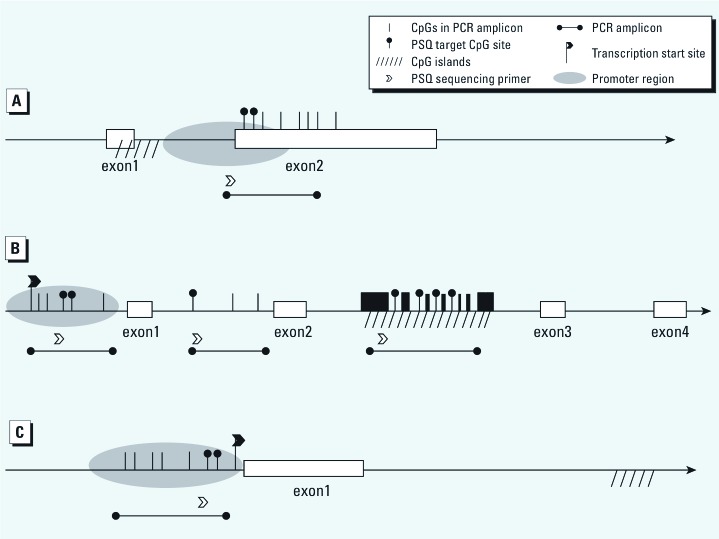
Schematic view of CpG locus depicts exon–intron structure and position of selected PCR amplicons for *NOS1* (*A*), *NOS2A* (*B*), and *NOS3* (*C*). The CpG positions were located at the following sites: chr12:117,769,133-117,769,145 for *NOS1*, chr17: 26,127,518-26,127,523 for non-CpG islands in the promoter of *NOS2A*, chr17: 26,126,265-26,126,267 for non-CpG island between exons 1 and 2 of *NOS2A*, chr17: 26,120,696-26,120,703 for CpG islands of *NOS2A*, chr7:150,690,770-150,690,776 for *NOS3*, according to assembly of GRCh37/hg19.

*Selection of CpG methylation loci.* We examined CpG loci located in *NOS1, NOS2A,* and *NOS3* (Chan GC et al. 2005; Chan Y et al. 2004; Frain et al. 1989; Shi et al. 1998; Tarantini et al. 2009; Wei et al. 2002) (Figure 1). Two CpG loci in a nuclear hormone receptor (NHR) regulatory sequence in exon 2 of *NOS1* were chosen for analysis, based on evidence that this NHR site contributes to the regulation of *NOS1* transcription (Wei et al. 2002). Seven CpG sites in three regions within the *NOS2A* gene were selected for DNA methylation analysis. Two loci were chosen in a non-CpG island region (positions 1 and 2) of the promoter because the promoter was previously shown to be inversely related to *iNOS* (alias: *NOS2A*) mRNA expression (Chan GC et al. 2005). A third CpG site located in a non-CpG island between exon 1 and exon 2 (position 3) was chosen to correspond to a previously investigated site by Tarantini et al. (2009). The third region included four CpG sites (positions 4–7) in a locus located in a CpG island that was chosen because it spans transcription factor binding sites conserved in the mammal alignment (http://genome.ucsc.edu/) but whose methylation status had not previously been investigated. For *NOS3*, we examined two CpG loci located in the positive regulatory domain in the proximal promoter, which has a binding affinity with Sp1/Sp3, an important transcription factor involved in *NOS3* transcription (Karantzoulis-Fegaras et al. 1999).

Polymerase chain reaction (PCR) primers targeting these loci were developed using MethPrimer software (Li and Dahiya 2002). Primers were designed not to overlap with any repeated elements and/or single-nucleotide polymorphism (SNP) sites and the specificity of the primer sequence was confirmed using *in silico* PCR [see Supplemental Material, Table S1 (http://dx.doi.org/10.1289/ehp.1104439)].

*DNA methylation.* Laboratory personnel performing DNA methylation analysis were blinded to study subject information. Bisulfite conversion of 1 μg of genomic DNA extracted from buccal mucosal cells was performed with the EZ-96 DNA Methylation-Gold Kit™ (Zymo Research, Orange, CA), according to the manufacturer’s recommended protocol. Final elution was performed with 40 μL M-elution buffer. Bisulfite-converted DNA was stored at –70°C until further use. Methylation analyses were performed by bisulfite-PCR. Pyrosequencing assays were performed using the HotMaster Mix (Eppendorf, Hamburg, Germany) and the Pyrosequencing (PSQ) HS 96 Pyrosequencing System (Biotage AB, Uppsala, Sweden) (Jirtle and Skinner 2007) as described in previous work (Byun et al. 2009). Hct116 cell line DNA was used as control DNA and placed on each of 10 plates run on the PSQ. Percent coefficient of variation across the plates ranged from 2.1% to 5.5% for *NOS2A* and 1.1% for *NOS3*. The output from Pyrosequencing is reported as a percent of DNA methylation at each CpG locus. As a quality control check to estimate the bisulfite conversion efficiency, we placed duplicate genomic DNA samples on each bisulfite conversion plate to estimate the internal plate variation of bisulfite conversion and the Pyrosequencing reaction. Conversion efficiency was > 95%. We also added universal PCR products amplified from cell-line DNA on each Pyrosequencing plate to check the run-to-run and plate-to-plate variation in performing Pyrosequencing reactions. In addition, the pyrogram peak pattern from every sample was checked to confirm the quality of reaction.

*Air pollutants.* Air pollution data were obtained from central monitoring sites in each study community operated by local air pollution control agencies in conformance with U.S. Environmental Protection Agency (EPA) monitoring requirements (U.S. EPA 2012). Each community contained a single central-site monitor. At each monitoring site, 24-hr average measurements PM_2.5_ were obtained daily or every third day. In addition, hourly PM_2.5_ measurements were collected at selected community air monitoring sites. Continuous hourly average measurements were made for PM_10_. When pollution data were not available for certain days, the gaps were filled by using data from nearby monitors provided that the monitors were not more than 7 km apart and the measurements from the monitors were reasonably well correlated (0.5 < *r*^2^ < 0.95, depending on site and season) with each other. Daily 24-hr averages of PM_2.5_ and PM_10_ were extracted for the buccal cell collection date to calculate cumulative average exposure levels 7 days, 1 month, 6 months, and 1 year before the date of DNA collection.

*Assessment of covariates.* Race/ethnicity, physician diagnosis of asthma, current wheeze, allergy, annual family income, parental education, and exposure to *in utero* and secondhand tobacco smoke (SHS) were obtained through annual written questionnaires completed by the parents. Children were considered to have asthma if their parents reported on the questionnaire that their child had ever been physician-diagnosed; questionnaires were collected at the school visit during which the child’s buccal cell sample was collected. Children were considered to have current wheeze if their parents reported on the same questionnaire that their child had wheezed in the previous 12 months. History of respiratory allergy was assessed by whether the child had a problem with sneezing or runny nose when he or she did not have a cold or the flu. Height and weight were measured on the day of collection. Age- and sex-specific percentiles based on the Centers for Disease Control and Prevention (2011) body mass index (BMI) growth charts were used to categorize BMI.

*Statistical analysis.* Descriptive analyses were first conducted to examine the distribution of DNA methylation in NOS genes and air pollutants by subject characteristics. Spearman correlations were used to study the pairwise correlations of percent methylation between different CpGs in the same gene. For genes with multiple CpG loci measured, percent DNA methylation at individual loci as well as average percent DNA methylation were analyzed. For the *NOS2A* CpG island, only the average of the percent methylation of four loci was evaluated.

To investigate the association between air pollutants and percent DNA methylation, we fitted beta regression models adjusted for age, sex, race/ethnicity, experimental plate (for Pyrosequencing reactions), town of residence, month of DNA collection, and parental education. Additional adjustment for asthma, allergy, and wheeze were evaluated, because presence of disease may affect methylation status. Asthma status was retained *a priori* in the model; however, further adjustment for allergy and wheeze status did not change the regression results and were dropped from final models. Beta regression was used to address the non-normal distribution of DNA methylation values, which are bounded by 0 and 1 and in many cases heavily skewed toward one end or the other (Ferrari and Cribari-Neto 2004). Beta regression requires the data to be between zero and one, therefore a shrinkage method was applied to force the zeros to be positive as follows: 0.999999 × (meth value – 0.5) + 0.5. Because beta regression is modeled on the logit scale, we transformed the results to a linear scale centered around the mean values for covariates in order to interpret the results as an absolute difference in percent methylation in the average individual for a given unit change in pollutant exposure. We scaled the results to a change in methylation per 5-µg/m^3^ increase in PM_2.5_ exposure because this was very close to the interquartile range in the population (4.8 µg/m^3^). One subject with an extremely high methylation value for *NOS2A* CpG island average was considered an outlier and removed from analyses.

We also used beta regression models to model differences in methylation levels by asthma and wheeze status in this population. *A priori* variables included for adjustment were age, sex, race/ethnicity, experimental plate, allergy, and town. Within asthmatics, stratified analyses by medication use, which can be used as an indicator of severity of asthma, were also conducted. For analyses restricted to asthmatics (*n* = 133), plate and town were dropped from models given the small sample size and large degrees of freedom required by these variables.

Sensitivity analyses were also conducted to evaluate whether the inclusion of children with asthma or wheeze influenced our results. Thus, the analyses of air pollutants and DNA methylation were rerun in a population in which children with asthma and wheeze were removed (*n* = 704).

All tests assumed a two-sided alternative hypothesis, a 0.05 significance level, and were conducted using SAS/STAT® software, version 9.2 (SAS Institute Inc., Cary, NC) and in the R programming language, version R2.12.2 (R Project for Statistical Computing, Vienna, Austria).

## Results

**Figure 2:**
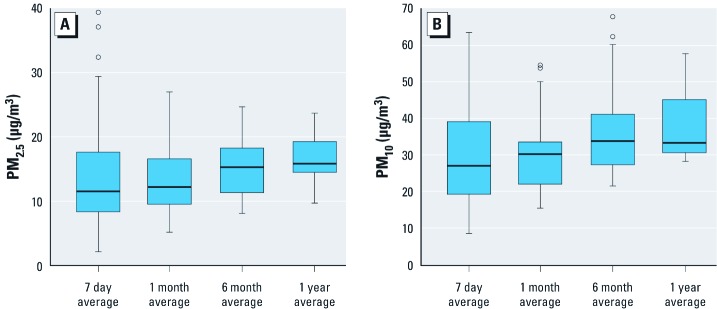
Distribution of cumulative average PM_2.5_ (*A*) and PM_10_ (*B*) (µg/m^3^) for 940 CHS participants by time period. Boxes extend from the 25th to the 75th percentile, horizontal bars represent the median, whiskers extend to the most extreme data point, which is no more than 1.5 times the interquartile range above and below the 75th and 25th percentiles, respectively, and outliers are represented as circles.

On average, children were 9 years old, two-thirds were of Hispanic white ethnicity, 14% had been diagnosed with asthma by the time of buccal cell collection, 11% had current wheezing reported by their parents, and 56% had a parentally-reported history of respiratory allergies (Table 1). Cumulative average PM_2.5_ and PM_10_ were right-skewed, median PM_2.5_ ranged from 11.5 to 15.8 µg/m^3^, and median PM_10_ ranged from 27.0 to 33.8 µg/m^3^ (Figure 2). Distributions of cumulative averages are shown in Supplemental Material, Table S2 (http://dx.doi.org/10.1289/ehp.1104439), and the correlations for PM_2.5_ and PM_10_ are shown in Supplemental Material, Table S3.

**Table 1:** Descriptive characteristics of the 940 selected CHS participants.

Characteristic	n (%)
Male	451 (48.0)
Race/ethnicity	
Hispanic white	607 (64.6)
Non-Hispanic white	333 (35.4)
Exposed to maternal smoking in utero	51 (5.6)
No. missing	30
Exposure to paternal smoking in utero	113 (12.5)
No. missing	38
Exposure to secondhand smoke	31 (3.5)
No. missing	49
Ever diagnosed with asthma	133 (14.2)
Current wheezea	96 (10.9)
No. missing	55
History of respiratory allergy	522 (55.6)
No. missing	1
Use of asthma medication in previous 12 months	88 (10.0)
No. missing	59
Annual family income	
≤ $14,999	126 (15.9)
$15,000–$49,999	240 (30.3)
≥ $50,000	425 (53.7)
No. missing	149
Parent/guardian education	
Less than 12th grade	193 (21.6)
Completed grade 12	163 (18.2)
Some college or technical school	302 (33.7)
Completed 4 years of college	125 (14.0)
Some graduate training	112 (12.5)
No. missing	45
Age (years) (mean ± SD)	9.3 ± 1.1
aAny wheeze in previous 12 months.	

Distribution of percent methylation of NOS loci is shown in Table 2. In general, the non-CpG island loci in *NOS1*, *NOS2A*, and *NOS3* were methylated > 75%. The proportion of methylation at the CpG locus in *NOS2A* position 3 (located in a non-CpG island between exon 1 and 2) was 51%, whereas CpG island loci in *NOS2A* were largely unmethylated (< 2.5%). Within each gene, the multiple CpG loci measured were not strongly correlated, with correlation coefficients < 0.5 for all but *NOS1* positions 1 and 3 [see Supplemental Material, Tables S4 and S5 (http://dx.doi.org/10.1289/ehp.1104439)].

**Table 2:** Distribution of mean percent methylation of CpG loci in *NOS* genes.

Gene loci	Locationa	nb	Mean ± SD		IQR	25th	Median	75th	Min	Max
NOS1																			
Position 1		Non-island		905		68.7	± 6.5		8.8		64.3		68.9		73.1		41.6		87.2
Position 2		Non-island		894		89.7	± 4.1		5.3		87.1		89.9		92.4		71.0		100.0
Position 3		Non-island		884		62.3	± 6.9		8.2		58.3		62.3		66.5		12.8		89.4
Averagec		Non-island		908		73.6	± 4.9		6.4		70.4		73.6		76.7		57.8		89.0
NOS2A																			
Position 1		Non-island		896		91.7	± 2.5		2.8		90.5		92.0		93.3		77.9		97.4
Position 2		Non-island		892		97.0	± 3.3		4.6		95.5		97.8		100.0		79.0		100.0
Position 3		Non-island		916		51.3	± 4.3		5.8		48.5		51.5		54.2		30.5		63.3
Position 4		Island		940		2.1	± 3.1		1.7		0.7		1.4		2.4		0.0		46.2
Position 5		Island		939		1.8	± 3.2		2.0		0.0		1.1		2.0		0.0		48.7
Position 6		Island		939		1.0	± 2.4		1.1		0.0		0.5		1.1		0.0		42.9
Position 7		Island		940		1.1	± 2.2		1.2		0.0		0.7		1.2		0.0		38.9
Promoter averageb		Non-island		896		94.3	± 2.6		3.0		93.2		94.8		96.2		80.5		98.7
CpG island averageb		Island		940		1.5	± 2.5		0.9		0.6		1.0		1.5		0.0		44.2
NOS3																			
Position 1		Non-island		914		89.2	± 7.0		7.3		86.2		90.5		93.5		43.1		100.0
Position 2		Non-island		914		90.6	± 6.0		6.3		88.1		91.9		94.4		47.3		100.0
Averageb		Non-island		914		89.9	± 5.4		6.0		87.4		90.9		93.4		58.3		100.0
Abbreviations: IQR, interquartile range; Max, maximum; Min, minimum. 25th and 75th are percentiles. aCpG islands are genomic regions 300–3,000 base pairs in length that contain a high frequency of CpG sites. bSample size varies as a result of quality control screening for Pyrosequencing reactions. cAverage methylation of multiple positions within the gene was used.																			

PM_2.5_ was negatively associated with percent DNA methylation in the non-CpG island regions of *NOS2A* (Table 3). In beta regression models, cumulative average PM_2.5_ exposure windows were significantly associated with lower percent methylation in positions 1 and 2, although the associations for position 1 had smaller *p*-values than for other positions. A 5-µg/m^3^ increase in acute (7 day, 1 month) and long-term (6 months, 1 year) cumulative averages of PM_2.5_ was associated with a 0.2% [95% confidence interval (CI): –0.32, –0.07] to 1% (95% CI: –1.61, –0.56) lower DNA methylation at position 1. In addition, cumulative averages of PM_2.5_ were associated with lower methylation of *NOS2A* at position 2 for 1-month and 1-year averages and at position 3 for 7-day and 6-month averages. In contrast, PM_2.5_ exposure was associated with small but significantly higher in percent DNA methylation of the average of positions 4–7 in the *NOS2A* CpG island region (Table 3).

**Table 3:** The difference in percent DNA methylation in *NOS2A* and *NOS3* per 5‑µg/m^3^ change in cumulative PM_2.5_ exposure, using beta regression.^a^

Average PM2.5 exposure						
Locus		Association	7 day	1 month	6 month	1 year
NOS2A Non-CpG island											
	Position 1		Difference in % methylationb		–0.20		–0.39		–0.77		–1.08
			95% CI		(–0.32, –0.07)		(–0.66, –0.11)		(–1.16, –0.39)		(–1.61, –0.56)
			n		896		896		896		801
	Position 2		Difference in % methylationb		–0.06		–0.34		–0.31		–0.58
			95% CI		(–0.18, 0.06)		(–0.62, –0.05)		(–0.70, 0.07)		(–1.13, –0.02)
			n		892		892		892		797
	Position 3		Difference in % methylationb		–0.34		–0.43		–0.89		–0.71
			95% CI		(–0.57, –0.11)		(–0.94, 0.08)		(–1.57, –0.21)		(–1.60, 0.18)
			n		916		916		916		821
NOS2A CpG islandc											
	Average of Position 4–7		Difference in % methylationb		0.07		0.12		0.21		0.33
			95% CI		(–0.001, 0.14)		(–0.04, 0.28)		(–0.02, 0.43)		(0.01, 0.65)
			n		939		939		939		842
NOS3											
	Position 1		Difference in % methylationb		0.56		1.13		1.77		2.11
			95% CI		(0.13, 0.99)		(0.24, 2.02)		(0.67, 2.87)		(0.72, 3.50)
			n		914		914		914		818
	Position 2		Difference in % methylationb		0.46		0.61		1.90		2.76
			95% CI		(0.09, 0.83)		(–0.17, 1.39)		(1.02, 2.79)		(1.77, 3.75)
			n		914		914		914		818
aBeta regression coefficients were transformed to a linear scale to reflect a change in methylation in response to 5‑µg/m3 increase relative to the mean pollutant level. bAll models were adjusted for age, sex, ethnicity, plate, month, town, and parental education and asthma status. cOne subject with extremely high methylation value was considered an outlier and removed from analyses.											

Because these analyses were conducted in all children, including those with a diagnosis of asthma or wheeze, we conducted a sensitivity analysis in which we estimated the associations between PM_2.5_ and *NOS2A* methylation in a population restricted to children without diagnosis of asthma or wheeze (*n* = 720). Associations were consistent with those for all children combined [see Supplemental Material, Table S6 (http://dx.doi.org/10.1289/ehp.1104439)].

PM_2.5_ was also positively associated with percent DNA methylation in both positions measured in *NOS3*. A 5-µg/m^3^ increase in acute (7 day) and long-term (1 year) cumulative average PM_2.5_ levels was associated with 0.6% (95% CI: 0.13, 0.99) to 2.8% (95% CI: 1.77, 3.75) higher absolute level of percent DNA methylation in the two loci (Table 3). PM_10_ showed associations with DNA methylation in the NOS genes that were smaller in magnitude compared with PM_2.5_ and often lacked statistical significance [see Supplemental Material, Table S7 (http://dx.doi.org/10.1289/ehp.1104439)].

DNA methylation in *NOS2A* was investigated with respect to current childhood wheeze. Children with current wheeze had 0.21% (95% CI: –0.22, 0.64) higher methylation in *NOS2A* position 1, 0.39% (95% CI: –0.84, 0.07) lower methylation in the *NOS2A* position 2 and 0.25% (95% CI: –1.07, 0.58) lower methylation in the *NOS2A* position 3 compared with children without wheeze. No direct association between DNA methylation and asthma overall was observed. However, among 133 children with asthma, children with current wheeze only (*n* = 54) and children taking asthma medications (*n* = 66) had lower DNA methylation in the *NOS2A* promoter region than did asthmatic children without a recent history of wheeze or medication use, respectively. Children using asthma medication had 0.49% (95% CI: –1.42, 0.45), 1.72% (95% CI: –2.77, –0.67), and 1.37% (95% CI: –2.79, 0.06) lower DNA methylation in *NOS2A* positions 1, 2, and 3, respectively, compared with asthmatic children not taking medication. Asthmatic children with current wheeze had 0.28% (95% CI: –1.22, 0.66), 0.90% (95% CI: –2.01, 0.20), and 0.93% (95% CI: –2.39, 0.54) lower DNA methylation in *NOS2A* positions 1, 2, and 3, respectively, compared with asthmatic children without wheeze.

Of the pollutants evaluated, PM_2.5_ was consistently and significantly associated with DNA methylation levels in NOS genes, primarily *NOS2A* and *NOS3*. PM_10_ showed similar associations with DNA methylation in these genes as compared with PM_2.5_ but the magnitude of association was weaker. No statistically significant associations were observed for *NOS1* (results not shown).

## Discussion

In this study, PM_2.5_ was associated with differences in level of percent DNA methylation in NOS genes, suggesting that NO homeostasis may be influenced by air pollution. NOS DNA methylation was also associated with wheeze and medication use, a proxy for active disease, in children with asthma.

Particulate matter is associated with increased expression of iNOS (Ulrich et al. 2002). A decrease in DNA methylation in the promoter of *NOS2A*—the gene encoding iNOS—that increases transcription of the gene is one hypothesis to explain such an observed association. A recent study by Tarantini et al. provided preliminary evidence in support of such a theory (Baccarelli et al. 2009; Tarantini et al. 2009). In that study, *NOS2A* DNA methylation level decreased after a 3-day shift in a steel mill; however, methylation was not associated with measured PM_10_ levels in the workers (Baccarelli et al. 2009; Tarantini et al. 2009). In the present study, we estimated short- and long-term effects of both PM_10_ and PM_2.5_ on DNA methylation of several CpG loci in promoter and nonpromoter regions of *NOS2A*. PM_2.5_ was more strongly associated with lower *NOS2A* promoter methylation than PM_10_. We observed a significant association of 7-day cumulative average PM_2.5_ with percent DNA methylation in the same locus that Tarantini et al. (2009) evaluated (*NOS2A* Position 3), although stronger associations were observed for short and long-term cumulative averages at the two *NOS2A* promoter CpG loci more proximal to the transcription start site.

Interestingly, although PM_2.5_ was associated with lower methylation in the *NOS2A* promoter, it was also associated with higher methylation in the CpG island in the body of the gene. Regulation of iNOS activity is complex, and the inducibility of iNOS by cytokines is known to involve DNA methylation, histone H3 lysine 9 methylation, and MeCP2 binding at the promoter (Chan GC et al. 2005). Our observation that PM_2.5_ was associated with higher DNA methylation in the promoter suggests that iNOS expression might also have been increased in association with PM_2.5_ exposure. However, little is known about the functionality of DNA methylation in the downstream CpG island, and recent evidence suggests that methylation in CpG islands can in some cases be associated with increased expression rather than decreased expression (Bogdanovic et al. 2011).

Regulation of *NOS3* is similar to that of *NOS2A* in that increased DNA methylation in the promoter is associated with decreased promoter activity (Chan Y et al. 2004). However, the association between PM_2.5_ and *NOS3* methylation that we observed was opposite the association between PM_2.5_ and the *NOS2A* promoter. Air pollution was associated with higher DNA methylation in the *NOS3* promoter, which should lead to reduced transcriptional activity and lower NO production. This is consistent with evidence that cigarette smoke extract reduces *NOS3* activity, protein, and mRNA levels in pulmonary artery endothelial cells (Su et al. 1998). Moreover, Ten Broeke et al. (2006) observed that while iNOS expression was associated with increased Fe_NO_ in a mouse model of asthma, eNOS overexpression attenuated airway inflammation and hyperresponsiveness in allergic asthma. PM_2.5_-induced increases in DNA methylation of *NOS3* may contribute to exacerbation of inflammation and hyperresponsiveness via this mechanism. The pathophysiology of asthma may be determined by a delicate balancing of the production of NO by NOS isoforms. This theory is further supported by experimental evidence that demonstrated a simultaneous increase in iNOS expression and decrease in eNOS expression in response to endotoxin challenge in intact rat lungs (Ermert et al. 2002).

Among all children, children with wheeze had lower levels of DNA methylation in two of the three loci in the *NOS2A* promoter than did children without wheeze. Among children with asthma, *NOS2A* methylation was lower among those with active wheeze and those currently taking medication compared with asthmatic children without wheeze or medication use, respectively. One explanation for these results is that NO production may be up-regulated via an epigenetic mechanism in allergic airway disease. This explanation would be consistent with findings previously reported on DNA methylation in *ARG1* and *ARG2* genes, also key players in NO homeostasis, in relation to exhaled nitric oxide (Breton et al. 2011). However, these results are based on a small sample size and the conclusions should be interpreted with caution.

Given that PM_2.5_ was associated with lower DNA methylation in the *NOS2A* promoter, epigenetic regulation of *NOS2A* may provide one biological mechanism by which PM_2.5_ affects respiratory health outcomes in children. Epigenetic regulation of other genes has also been implicated in association with air pollution and asthma. Children in a community with high levels of ambient air pollution (Fresno, CA) had increased methylation of the *FOXP3* locus, a locus important in Treg-cell function and asthma morbidity, compared with children residing in a low-pollution community (Palo Alto, CA) (Nadeau et al. 2010). In a cohort of children living in New York City, exposure to PAHs was associated with DNA methylation in the *ACSL3* gene, a gene associated with asthma symptoms in children (Perera et al. 2009). Emerging evidence suggests a link between air pollution exposures, alterations in DNA methylation levels and downstream health outcomes.

Although associations between air pollution and iNOS methylation have been observed across studies, the magnitudes of associations for single CpG methylation loci are small and thus their biological relevance is unclear. Nevertheless, we previously reported that in children with the highest 10th percentile of iNOS methylation (> 56.6%), PM_2.5_ exposure was significantly associated with higher Fe_NO_ levels, whereas no association was seen at lower methylation levels (Salam et al. 2012). *NOS2A* genetic and epigenetic variations and short-term PM_2.5_ acted synergistically in association with Fe_NO_ levels in our population, suggesting that a small change in DNA methylation at one CpG locus may have functional significance. However, regulation of Fe_NO_ is complex and future studies warrant the joint evaluation of genetic and epigenetic variations and air pollution exposure on phenotype expression.

We have observed particulate matter-associated differences in DNA methylation in buccal cells. Our associations are similar in direction of effect and magnitude to those of Tarantini et al. (2009) despite the fact that different cell types were used. However, the implications of DNA methylation changes for health outcomes are far from clear and are likely to differ by cell type and disease pathophysiology. For example, Chan GC et al. (2005) demonstrated variable levels of inducibility of iNOS dependent on cell type investigated. Ideally, methylation changes in NOS genes should be evaluated in the most appropriate tissue for the outcome of interest in order to study functional consequences.

In evaluating childhood wheeze and asthma, buccal mucosal cells—an aerodigestive tract epithelium—were used for DNA methylation analysis. Previous studies have demonstrated that buccal epithelium can serve as a surrogate tissue for the lung when measuring DNA methylation (Bhutani et al. 2008). In addition, studies comparing expression profiles in buccal, bronchial, and nasal epithelial cells in response to tobacco smoke have demonstrated striking similarities, providing further support for use of buccal cells as a useful surrogate for respiratory tract cells (Boyle et al. 2010; Sridhar et al. 2008). However, buccal cells are a mixed population of epithelial cells and leukocytes. Thus, a shift in cell populations caused by air pollutant exposure, rather than by differences in methylation, could explain our observed results.

Buccal-cell DNA was collected after diagnosis of asthma and wheeze, so we cannot address whether DNA methylation in NOS genes precedes or is the result of childhood respiratory disease. Similarly, we cannot rule out the possibility that asthma medications may have altered DNA methylation in the asthmatic children. However, the observed trends showing that children with wheeze had intermediate levels of DNA methylation, compared with healthy children and children with active asthma, support the conclusion that DNA methylation levels in NOS genes may play a role in asthma pathogenesis. Further investigation in a longitudinal study design that collects DNA samples at or near birth would clarify these lingering concerns and is currently underway.

We evaluated four cumulative average exposure levels of PM_2.5_ and PM_10_ before the date of DNA collection. Our choice to evaluate short-term cumulative averages was based on previous evidence of an association between cumulative pollutant exposure and Fe_NO_, the product of the genetic pathway of interest (Berhane et al. 2011), and on the knowledge that the half-life of buccal cells is only a few weeks. However, long-term exposure effects on DNA methylation may also be possible in buccal cells if exposure affects the basal or progenitor cells from which the surface epithelium is formed, or if methylation marks, once altered, are heritably passed on from one generation of cells to the next. The shorter cumulative averages are more likely to have increased measurement error in exposure assessment which may explain the weaker statistical results. The chronic exposure estimates are more robust and representative of typical and steady state exposures.

Twelve distinct CpG loci in three NOS genes, and two air pollutants, were evaluated in this study. Given the multiple tests that were conducted, some associations may be attributable to chance. Last, parental report of physician-diagnosed asthma was used in our study, and concern has been raised that parental report might not reflect physician diagnosis. To investigate this potential bias, we reviewed medical records of 172 children with asthma and found that 95.9% had a definite or probable diagnosis of asthma (Salam et al. 2007).

## Conclusion

In this study, we provide evidence that DNA methylation levels in NOS genes vary by PM_2.5_ exposure levels and possibly wheeze during childhood. These results add to a small but growing body of literature suggesting epigenetic regulation of certain genes as potentially important biological mechanisms underlying the health effects of air pollution exposures.

## Supplemental Material

(78 MB) PDF
