# Supplementary material for: Particulate Matter, DNA Methylation in Nitric Oxide Synthase, and Childhood Respiratory Disease
Source: Environ Health Perspect. 2012 May 16;120(9):1320–6. doi: 10.1289/ehp.1104439 (PMC3440108; doi:10.1289/ehp.1104439)
Supplement: (78 MB) PDF [file ehp.1104439.s001.pdf]

**Supplemental Material**

# Particulate Matter, DNA Methylation in Nitric Oxide Synthase, and Childhood Respiratory Disease

Carrie V. Breton[1], Muhammad T. Salam[1], Xinhui Wang[1], Hyang-Min Byun[2], Kimberly D. Siegmund[1] and Frank D. Gilliland[1]

[1]Department of Preventive Medicine, Keck School of Medicine, University of Southern California, 1540 Alcazar Street, CHP 236, Los Angeles, CA

[2] Exposure, Epidemiology & Risk Program, Harvard School of Public Health, Boston, MA

Contents

Supplemental Material, Table 1....................................................................................2

Supplemental Material, Table 2....................................................................................3

Supplemental Material, Table 3 ....................................................................................4

Supplemental Material, Table 4....................................................................................5

Supplemental Material, Table 5....................................................................................6

Supplemental Material, Table 6....................................................................................7

Supplemental Material, Table 7....................................................................................8

**Tables**

**Supplemental Material, Table S1. Primer sequences and reaction conditions for *NOS* genes**

| | **Primer[a]** | **Sequence** | **Annealing Temp** | **PCR size(bp)** |
|---|---|---|---|---|
| NOS1 | PCR Forward | NOS1-F:AGGTTGGTAATGAAGATATTTAGAGAATAG | 56.7$^{o}$C | 223 |
| | PCR Reverse | NOS1-R(biotin) : TCACCCACTCATAACTAATAACCC | | |
| | PSQ sequencing | NOS1-SP:TTTTAGGGATA | | |
| NOS2A | PCR Forward | iNOS(23151743)-F: AAAAATAATTTTTTGGATGGTATGG | TDOWN53 | 177 |
| | PCR Reverse | iNOS(23151567)-R(biotin): AAACTATCTAAAACTACCCAATCCC | | |
| | PSQ sequencing | iNOS(23151671)-SP:TTTATAATTTTGTAG | | |
| NOS2A | PCR Forward | iNOS(23150425)-F: TTAGGGTTAGGTAAAGGTATTTTTGTTT | TDOWN53 | 212 |
| | PCR Reverse | iNOS(23150214)-R(biotin): CAATTCTATAAAACCACCTAATAATCTTAA | | |
| | PSQ sequencing | iNOS(23150425)-SP: TTAGGGTTAGGTAAAGGTATTTTTGTTT | | |
| NOS2A | PCR Forward | iNOS(23145018)-F: GGAAGGTAGGGAAGGAGGGGTAGTT | TNCTD | 243 |
| | PCR Reverse | iNOS(23144776)-R(biotin): AAAAATCCTACAAAACAACCTACACAACC | | |
| | PSQ sequencing | iNOS(23144840)-SP: GAGGGGTTGGG | | |
| NOS3 | PCR Forward | NOS3-F: GGATATTTGGGTTTTTATTTA | TDOWN53 | 187 |
| | PCR Reverse | NOS3-R(biotin): CAATAAAAAAAAACTCTCCA | | |
| | PSQ sequencing | NOS3-SP: TGGGATAGGGG | | |

[a] Primers were designed using MethPrimer software, with parameters for selection including a product size of 100~300bp, 50~60oC for primer Tm, and a primer size of 20~30bp.

**Supplemental Material, Table S2. Distribution of $PM_{2.5}$ and $PM_{10}$ across 8 Southern California communities (N=940)**

| | $PM_{2.5}$ (μg/m$^3$) | | | | | $PM_{10}$ (μg/m$^3$) | | | | |
|---|---|---|---|---|---|---|---|---|---|---|
| **Cumulative average** | **Mean** | **Median** | **IQR** | **Min** | **Max** | **Mean** | **Median** | **IQR** | **Min** | **Max** |
| 7 day | 13.8 | 11.5 | 9.3 | 2.1 | 39.4 | 30.2 | 27.0 | 19.8 | 8.5 | 63.6 |
| 1 month | 13.5 | 12.2 | 7.1 | 5.2 | 26.9 | 29.4 | 30.2 | 11.4 | 15.5 | 54.6 |
| 6 month | 15.4 | 15.3 | 7 | 8.1 | 24.7 | 35.9 | 33.8 | 13.8 | 21.5 | 67.8 |
| 1 year | 16.7 | 15.8 | 4.8 | 9.7 | 23.7 | 38.3 | 33.2 | 14.6 | 28.2 | 57.7 |

**Supplemental Material, Table S3. Spearman correlation between cumulative average air pollutant exposures in 940 selected CHS participants**

| | 7 day $PM_{2.5}$ | 1 month $PM_{2.5}$ | 6 month $PM_{2.5}$ | 1 year $PM_{2.5}$[a] | 7 day $PM_{10}$ | 1 month $PM_{10}$ | 6 month $PM_{10}$ | 1 year $PM_{10}$[a] |
|---|---|---|---|---|---|---|---|---|
| 7 day $PM_{2.5}$ | 1 | 0.70 | 0.56 | 0.56 | 0.80 | 0.63 | 0.52 | 0.47 |
| 1 month $PM_{2.5}$ | | 1 | 0.80 | 0.73 | 0.56 | 0.81 | 0.71 | 0.68 |
| 6 month $PM_{2.5}$ | | | 1 | 0.93 | 0.38 | 0.59 | 0.85 | 0.75 |
| 1 year $PM_{2.5}$ | | | | 1 | 0.46 | 0.53 | 0.78 | 0.84 |
| 7 day $PM_{10}$ | | | | | 1 | 0.77 | 0.52 | 0.49 |
| 1 month $PM_{10}$ | | | | | | 1 | 0.75 | 0.63 |
| 6 month $PM_{10}$ | | | | | | | 1 | 0.86 |
| 1 year $PM_{10}$ | | | | | | | | 1 |

[a] sample size is 843 for 1 year cumulative exposures
p <0.0001 for all correlations

**Supplemental Material, Table S4. Spearman pairwise correlations for *NOS2A* CpG loci**

| | Position 1 | Position 2 | Position 3 | Position 4 | Position 5 | Position 6 | Position 7 |
|---|---|---|---|---|---|---|---|
| Position 1 | 1 | 0.46* | 0.14* | 0.06 | -0.06 | -0.09* | -0.02 |
| Position 2 | | 1 | 0.06* | 0.05 | 0.03 | -0.04 | -0.02 |
| Position 3 | | | 1 | 0.12* | 0.07* | -0.03 | 0.02 |
| Position 4 | | | | 1 | 0.34* | 0.20* | 0.25* |
| Position 5 | | | | | 1 | 0.30* | 0.42* |
| Position 6 | | | | | | 1 | 0.35* |
| Position 7 | | | | | | | 1 |

*p<0.05

**Supplemental Material, Table S5. Spearman pairwise correlations for *NOS1* and *NOS3* CpG loci**

| | | ***NOS1*** | | | ***NOS3*** | |
|---|---|---|---|---|---|---|
| | | **Position 1** | **Position 2** | **Position 3** | **Position 1** | **Position 2** |
| *NOS1* | Position 1 | 1 | 0.50* | 0.63* | -0.05 | -0.08* |
| | Position 2 | | 1 | 0.46* | -0.02 | -0.05 |
| | Position 3 | | | 1 | -0.08* | -0.10* |
| *NOS3* | Position 1 | | | | 1 | 0.41* |
| | Position 2 | | | | | 1 |

*p<0.05

**Supplemental Material, Table S6. The difference in % DNA methylation in *NOS2A* non-CpG island per 5 µg/m$^3$ change in cumulative $PM_{2.5}$ exposure restricted to a non-asthmatics and non-wheezers, using beta regression[a]**

| | Non-CpG island (promoter) | | | | | | Non CpG island (between exons 1 and 2) | | | CpG island[c] | | |
|---|---|---|---|---|---|---|---|---|---|---|---|---|
| | Position 1 | | | Position 2 | | | Position 3 | | | average of Position 4-7 | | |
| **Average $PM_{2.5}$ exposure** | **Difference in % methylation[b]** | **95%CI** | **N** | **Difference in % methylation[b]** | **95%CI** | **N** | **Difference in % methylation[b]** | **95%CI** | **N** | **Difference in % methylation[b]** | **95%CI** | **N** |
| 7 day | -0.26 | (-0.40, -0.12) | 689 | -0.01 | (-0.14, 0.13) | 686 | -0.38 | (-0.64, -0.12) | 704 | 0.09 | (0.01, 0.17) | 720 |
| 1 month | -0.44 | (-0.76, -0.12) | 689 | -0.15 | (-0.46, 0.16) | 686 | -0.31 | (-0.90, 0.28) | 704 | 0.18 | (-0.02, 0.37) | 720 |
| 6 month | -0.88 | (-1.32, -0.44) | 689 | -0.18 | (-0.57, 0.25) | 686 | -0.62 | (-1.39, 0.15) | 704 | 0.23 | (-0.03, 0.50) | 720 |
| 1 year | -1.27 | (-1.87, -0.67) | 612 | -0.51 | (-1.12, 0.11) | 609 | -0.39 | (-1.39, 0.61) | 626 | 0.40 | (0.02, 0.78) | 641 |

[a] Beta regression coefficients were transformed to a linear scale to reflect a change in methylation in response to 5 µg/m$^3$ increase relative to the mean pollutant level

[b] All models were adjusted for age, sex, ethnicity, plate, month, town, and parental education

[c] One subject with extremely high methylation value was considered an outlier and removed from analyses

**Supplemental Material, Table S7. The difference in % DNA methylation in *NOS2A* and *NOS3* per 5 μg/m$^3$ increase in cumulative $PM_{10}$ exposure, using beta regression**[a]

| Locus | | Association | Average $PM_{2.5}$ exposure | | | |
|---|---|---|---|---|---|---|
| | | | **7 day** | **1 month** | **6 month** | **1 year** |
| *NOS2A* Non-CpG island | Position 1 | Difference in % methylation[b] | -0.10 | -0.05 | -0.21 | -0.25 |
| | | 95% CI | (-0.18, -0.01) | (-0.18, 0.09) | (-0.38, -0.04) | (-0.59, 0.08) |
| | | N | 896 | 896 | 896 | 801 |
| | Position 2 | Difference in % methylation[b] | 0.06 | 0.15 | 0.07 | -0.21 |
| | | 95% CI | (-0.01, 0.14) | (0.03, 0.27) | (-0.08, 0.22) | (-0.54, 0.13) |
| | | N | 892 | 892 | 892 | 797 |
| | Position 3 | Difference in % methylation[†] | -0.11 | -0.09 | -0.47 | -0.23 |
| | | 95% CI | (-0.26, 0.05) | (-0.35, 0.17) | (-0.79, -0.15) | (-0.87, 0.40) |
| | | N | 916 | 916 | 916 | 821 |
| *NOS2A* CpG island [c] | Average of Position 4-7 | Difference in % methylation[b] | 0.01 | -0.01 | 0.04 | 0.20 |
| | | 95% CI | (-0.04, 0.05) | (-0.08, 0.07) | (-0.06, 0.13) | (-0.01, 0.41) |
| | | N | 939 | 939 | 939 | 842 |
| *NOS3* | Position 1 | Difference in % methylation[b] | 0.23 | 0.24 | 0.75 | 1.48 |
| | | 95% CI | (-0.06, 0.52) | (-0.25, 0.73) | (0.18, 1.32) | (0.40, 2.55) |
| | | N | 914 | 914 | 914 | 818 |
| | Position 2 | Difference in % methylation[b] | 0.20 | 0.01 | 0.68 | 1.44 |
| | | 95% CI | (-0.04, 0.44) | (-0.42, 0.43) | (0.19, 1.17) | (0.61, 2.27) |

| | | | | |
|---|---|---|---|---|
| N | 914 | 914 | 914 | 818 |

[a] Beta regression coefficients were transformed to a linear scale to reflect a change in methylation in response to 5 $\mu g/m^3$ increase relative to the mean pollutant level

[b] All models were adjusted for age, sex, ethnicity, plate, month, town, and parental education, and asthma status

[c] One subject with extremely high methylation value was considered an outlier and removed from analyses
